# Evaluation of the Sleep-Prolonging Effect of *Lagenaria vulgaris* and *Cucurbita pepo* Extracts on Pentobarbital-Induced Sleep and Possible Mechanisms of Action

**DOI:** 10.3390/medicina54040055

**Published:** 2018-07-31

**Authors:** Vafa Baradaran Rahimi, Vahid Reza Askari, Amineh Sadat Tajani, Azar Hosseini, Hassan Rakhshandeh

**Affiliations:** 1 Student Research Committee, Department of Pharmacology, Faculty of Medicine, Mashhad University of Medical Sciences, Mashhad 91778-99191, Iran; baradaranv941@mums.ac.ir; 2 Pharmacological Research Center of Medicinal Plants, Mashhad University of Medical Sciences, Mashhad 91778-99191, Iran; HoseiniAZ@mums.ac.ir; 3 Department of Pharmaceutical Control, School of Pharmacy, Mashhad University of Medical Sciences, Mashhad 91778-99191, Iran; TajaniA941@mums.ac.ir

**Keywords:** insomnia, *Lagenaria vulgaris*, *Cucurbita pepo*, flumazenil, naloxone

## Abstract

*Introduction:* Sleeplessness is the most common sleep disorder. In this study, the hypnotic effect of macerated (HAME) and soxhlet (HASE) extract of *Lagenaria vulgaris* (fruit and seed) and *Cucurbita pepo* (fruit) were studied in mice. *Methods:* Extracts and fractions were administered intra-peritoneally (i.p.) in mice 30 min before the sodium pentobarbital (30 mg/kg, i.p.). Moreover, the influence of flumazenil or naloxone on the hypnotic effects of the extract and its toxic effects were evaluated. *Results:* The HAME and HASE of *C. pepo* prolonged the pentobarbital-induced sleep duration at dose of 200 mg/kg. The HAME of *L. vulgaris* (fruit) at dose of 200 mg/kg increased the sleeping time. The HAME and HASE of *L. vulgaris* (seed) increased sleep duration at doses of 50 and 100 mg/kg. Besides, flumazenil (2 mg/kg) reversed the effects of both diazepam (*P* < 0.001 vs. diazepam group), 200 mg/kg of HAME of *C. pepo* and 50 mg/kg of HAME and HASE of *L. vulgaris* (seed). All fractions especially ethyl-acetate fraction (EAF) of *L. vulgaris* (seed) increased the sleep duration. Naloxone reversed the hypnotic effect of HAME and HASE of *L. vulgaris* (seed). The extracts showed no neurotoxic effects on PC12 and L929 cell lines. *Conclusion:* The results showed that *L. vulgaris* (seed and fruit) and *C. pepo* potentiated pentobarbital hypnosis without toxic influence. The hypnotic effects of *L. vulgaris* seed was greater than its fruit and *C. pepo*. The GABA and opioid receptors may play role in the sleep-induction of *L. vulgaris* seed.

## 1. Introduction

Sleep is a complex and physiological condition that plays and important role in our body’s function and health. Sleep is a vital process and is needed for various processes, such as learning, cellular repair and brain development. It can be impaired by certain factors including noise, stress and sickness [1,2]. It is estimated that more than 27% of people worldwide suffer from sleep disorders. Chronic sleep disorders lead to some health repercussions such as slower reactions, poor memorizing, emotional disturbances, and changes in immune response [3]. Clinically, sleep insufficiency leads to an increased risk of depression, obesity, dyslipidemia, hypertension and type 2 diabetes [4,5]. Different hypnotic drugs have been approved in order to treat insomnia, including benzodiazepine receptor (BZR) and melatonin receptor agonists, as well as histamine antagonists [6]. The benzodiazepine receptor agonists are the best choice and have been used for about 50 years [7]. However, it has been reported that they possess several side effects, including psychomotor retardation, memory impairment, paradoxical disinhibiting, depression and emotional blunting, tolerance and dependence and short-term withdrawal symptoms. These effects may be amplified in the elderly [8,9]. Therefore, it is still necessary to find new hypnotic drugs with fewer side effects and more effectiveness.

Herbal medicine has always been a good source for the development of new remedies. It has been demonstrated that some herbal agents are effective in ameliorating insomnia disorder including *Valeriana* spp. [10], *H. lupulus* [11], *Zizyphus jujuba* (sour date) [12], *V. officinalis* [13], *Passiflora incanata* (passionflower) [14], *Eschscholzia californica* (California poppy) [15], *Piper methysticum* [16] and *Lactuca sativa* [17]. Kaushik et al. suggested that triethylene glycol, an active component of *Withania somnifera*, propagates non-rapid eye movement (NREM) sleep in mice [18]. Furthermore, it has been demonstrated that octacosanol, an active compound of many foodstuffs, meaningfully elevates NREM sleep and mitigates sleep latency following stress-induced insomnia in mice. However, it did not improve sleep in normal mice [19].

*Lagenaria vulgaris* (*L. vulgaris* or *sicereria*) belongs to cucurbitaceae and is widely cultivated all over the world. In traditional medicine, it is used for the treatment of jaundice, diabetes, ulcer, piles, colitis, insanity, hypertension, congestive cardiac failure and skin diseases. It is also known as an emetic, purgative, cooling, sedative, anti-bilious and pectoral agent [20]. Recent studies have shown different properties of this plant such as analgesic, anti-inflammatory, anti-hyperlipidemic, diuretic, anthelmintic, anti-hepatotoxic, immuno-modulatory, anti-microbial and anti-oxidant activities [21]. *Cucurbita pepo* (*C. pepo*, green squash) belongs to cucurbitaceae and has some properties in Islamic references such as anti-depressant effect, anti-inflammation, jaundice and insomnia [22]. In folk medicine, *L. vulgaris* and *C. pepo* are most widely used for their sedative–hypnotic effect. However, there is no pharmacological evidence of the sedative–hypnotic effects of these macerated extracts. Therefore, this study is going to evaluate the sleep-prolonging effects and possible sleep mechanisms of *L. vulgaris* and *C. pepo* hydro-alcoholic extract.

## 2. Materials and Methods

### 2.1. Chemicals and Reagents

Dimethyl sulfoxide (DMSO, code D4540), penicillin-streptomycin (code P4333), sodium pentobarbital (code P3761), and 3-(4,5-Dimethyl-2-thiazolyl)-2,5-Diphenyl-2H-tetrazolium bromide (MTT, code M-5655) were purchased from Sigma (St. Louis, MO, USA). Diazepam was bought from Chemidarou Company (Iran). Dulbecco’s Modified Eagles Medium (DMEM, code 12800-082) and fetal bovine serum (FBS, code 10270-106) were obtained from Gibco Life Technologies (Grand Island, NY, USA).

### 2.2. Plant Collection and Extraction

*C. pepo* and *L. vulgaris* fruits were obtained from local markets in Mashhad, Iran, in the month of August. The fruits were completely washed with a safe reagent, sliced, dried in the shade and then powdered. Also, fresh seeds of *L. vulgaris* were detached from the fruits and subsequently triturated. After that, the hydro-alcoholic maceration extract (HAME) and the hydro-alcoholic soxhlet extract (HASE) were prepared as described previously [23,24]. The yields of the different extracts were HAME: *C. pepo* fruit = 14%, *L. vulgaris:* seed = 17% *w*/*w* and fruit = 21% *w*/*w*; HASE: *C. pepo* fruit = 19%, *L. vulgaris:* seed = 21% *w*/*w*, fruit = 27% *w*/*w*. Then, ethyl acetate (EAF), N-butanol (NBF) and the water fraction (WF) were prepared using solvent–solvent extraction [25]. All of the fractions were kept at −20 °C until use. The WF was dissolved in saline and the EAF and NBF were dissolved in distilled water containing 1% DMSO.

### 2.3. Animals

Male albino mice weighing 25–35 g with an age of 4 weeks were used in this study. Before starting our experiments, the mice were maintained in separated standard cages, in silence and in a ventilated laboratory with 12 h cycles of light/night and humidity of 61 ± 3% at 22 ± 2 °C temperature. Also, they were allowed free access to standard lab chow and water. The study was executed in accordance with ethical guidelines approved by the Animal Care Use Committee of Mashhad University of Medical Sciences.

### 2.4. Sleep Induction Protocol

The animals were given a single dose of vehicle, diazepam and extracts intra-peritoneally (i.p.). After 30 min, pentobarbital (30 mg/kg body weight i.p.) was injected to induce sleep. For evaluation of the sleep duration, the mice were considered asleep if they stayed immobile and lost their righting reflex when positioned on their back. The time interval between the pentobarbital injection and onset of sleep was recorded as sleep latency. Mice were randomly divided into 20 groups, each consisting of 8 mice. Saline was given i.p. as a negative control and diazepam was used as a positive control (3 mg/kg body weight i.p.). Other groups were treated with HAME and HASE (200, 100, 50 mg/kg body weight). To assess the sleep mechanism, flumazenil (1 mg/kg i.p.), naloxone (5 mg/kg i.p.) and the combination of naloxone and flumazenil was administrated 30 min before diazepam, 200 mg/kg HASE *C. pepo*, 200 mg/kg HAME *L. vulgaris* and 50 mg/kg HASE *L. vulgaris* (best answer of each type of extract) [2].

### 2.5. Median Lethal Dose (LD_50_) Determination

Nineteen groups, each containing 2 mice were used for the determination of the LD_50_ of the sample HAME and HASE. Groups 1–18 were injected i.p. with 25, 50, 100, 200, 400, 800, 1600, 3200 and 6400 mg/kg of HAME and HASE of samples and group 19 received saline as a vehicle. The mortality rate was observed and recorded for a 24 h period. The highest dose that did not kill any mice and the lowest dose which led to the death of one animal were recorded. The mean of these two doses was considered as the median lethal dose [26].

### 2.6. Cytotoxicity and Neurotoxicity Assessment

The possible cytotoxicity of the extracts which had a best result in terms of prolonging sleep was tested on PC12 and L929 cell lines. PC12 cells were used as an in-vitro model to evaluate the neuro-protective or neurotoxic activity. L929 cells are considered to be a standard cell line for cytotoxicity assays according to US Pharmacopeia and are frequently used for testing the possible toxic effects of materials.

The cells were seeded in 96-well plates overnight in DMEM and supplemented with 10% FBS, penicillin (100 units/mL) and streptomycin (100 µg/mL). Then, the culture medium was changed to fresh medium, containing vehicle (DMSO 1%), HAME or HASE (50, 100, 200, 400 and 800 µg/mL). The cells were incubated for 24 h at 37 °C in an atmosphere of 5% CO_2_. Then, cell proliferation was evaluated using MTT assay as previously described [2,27]. Briefly, the MTT solution was added to the culture medium to make a final concentration of 0.5 mg/mL and incubated for 2 h. The optical density of the dye was measured at 545 nm using a StatFAX2100 ELISA reader (awareness Inc., Waltham, MA, USA).

### 2.7. Statistics

All values are expressed as mean ± SD. The normality of data distribution was assessed using the Kolmogorov–Smirnov normality test. After that, statistical analysis was performed using one way analysis of variance (ANOVA), followed by the Tukey–Kramer post hoc test using the GraphPad Prism^®^ (version 6.01, Graph Pad Software Inc., La Jolla, CA, USA) software package. Significant difference was set at *P* < 0.05.

### 2.8. Ethical Disclosures 

The study was executed in accordance with ethical guidelines approved by the Animal Care Use Committee of Mashhad University of Medical Sciences (IR.MUMS.fmd 901176) on 11 April 2012.

## 3. Results

### 3.1. Effect of C. Pepo on Sleep Duration

**Figure 1:**
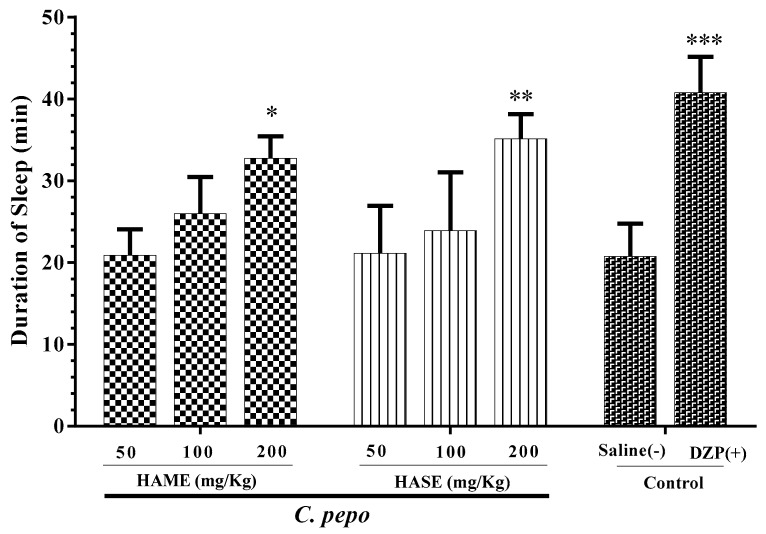
Effect of *C. pepo* extracts on sleep duration in mice. The animals were treated with saline, diazepam (DZP, 3 mg/kg), Hydro-alcoholic maceration extract (HAME) or hydro-alcoholic soxhlet extract (HASE), 30 min before administration of pentobarbital (30 mg/kg, i.p.). Data are expressed as mean ± SD (*n* = 8). * *P* < 0.05, ** *P* < 0.01 and *** *P* < 0.001 vs. saline. HAME, HASE, ethyl acetate fraction (EAF), N-butanol fraction (NBF), and the water fraction (WF).

Sleep duration in different groups of HAME and diazepam were compared to saline as the control group (Figure 1). The extract significantly increased sleep duration at a dose of 200 mg/kg (*P* < 0.05, Figure 1), while other doses (50 and 100 mg/kg) did not increase sleep duration. The HASE group was similar to HAME. HASE increased sleep duration at a dose of 200 mg/kg significantly (*P* < 0.01, Figure 1). A dose of 100 mg/kg increased sleep duration, but the result was not significant (Figure 1).

### 3.2. Effect of Fruit Extracts of L. vulgaris on Sleep Duration

**Figure 2:**
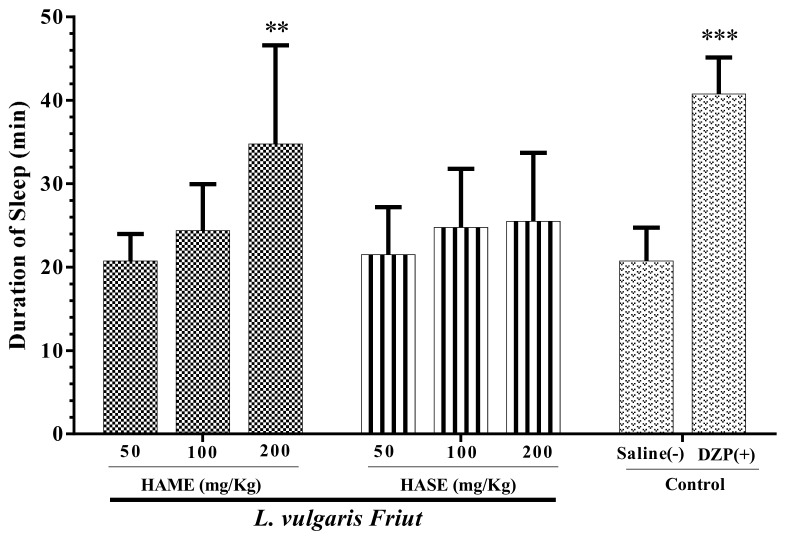
Effect of *L. vulgaris* fruit extracts on sleep duration in mice. The animals were treated with saline, diazepam (DZP, 3 mg/kg), HAME or HASE 30 min before administration of pentobarbital (30 mg/kg, i.p.). Data are expressed as mean ± SD (*n* = 8). ** *P* < 0.01 and *** *P* < 0.001 vs. saline. Hydro-alcoholic maceration extract (HAME), hydro-alcoholic soxhlet extract (HASE), ethyl acetate fraction (EAF), N-butanol fraction (NBF), and the water fraction (WF).

Figure 2 shows that all the doses of fruit extracts did not significantly increase the sleep duration time, except 200 mg/kg of HAME (*P* < 0.01). The doses 200 and 100 mg/kg of HASE and 100 mg/kg of HAME prolonged sleep duration, but the results were not significant (Figure 2).

### 3.3. Effect of Seed Extracts of L. Vulgaris on Sleep Duration

**Figure 3:**
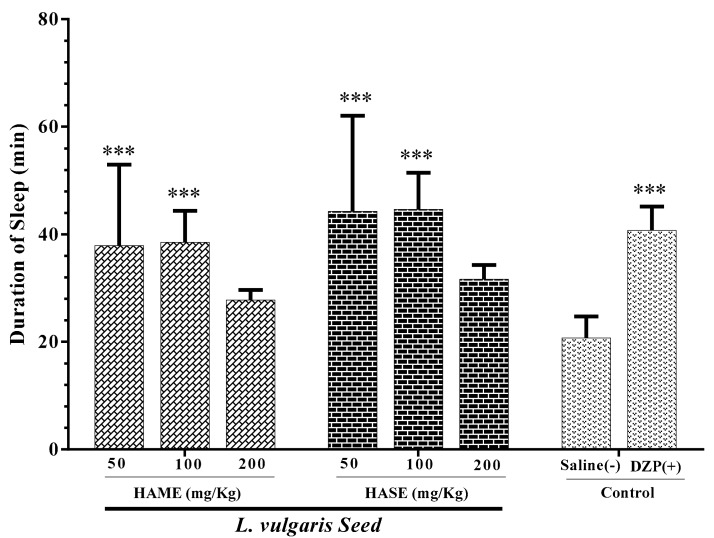
Effect of *L. vulgaris* seed extracts on sleep duration in mice. The animals were treated with saline, diazepam (DZP, 3 mg/kg), HAME or HASE 30 min before administration of pentobarbital (30 mg/kg, i.p.). Data are expressed as mean ± SD (*n* = 8). *** *P* < 0.001 vs. saline. Hydro-alcoholic maceration extract (HAME), hydro-alcoholic soxhlet extract (HASE), ethyl acetate fraction (EAF), N-butanol fraction (NBF), and the water fraction (WF).

The effect of the HASE and HAME of *L. vulgaris* seeds (50 and 100 mg/kg) on the sleep duration time was markedly propagated in comparison to the NS group (*P* < 0.001 for all cases, Figure 3).

**Figure 4:**
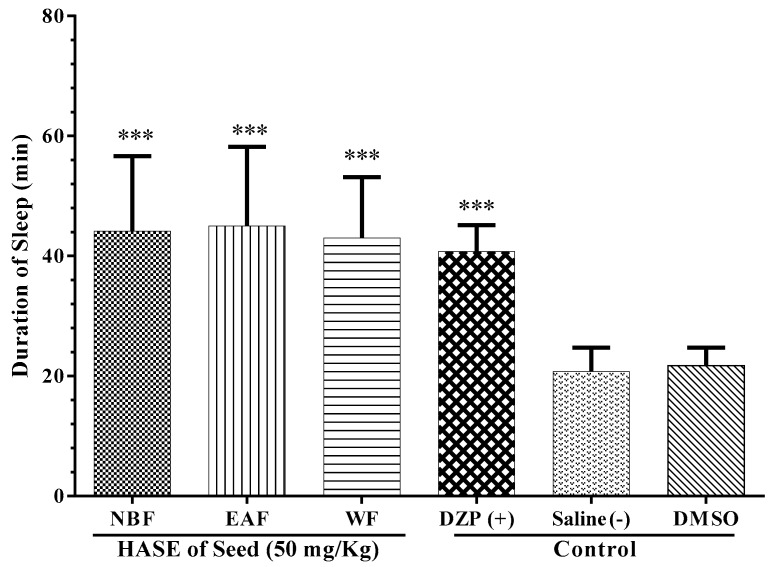
Effects of seed fractions of *L. vulgaris* on sleep duration in mice. The animals were treated with 10% DMSO, saline, diazepam (DZP, 3 mg/kg) or seeds fractions of HASE including: water fraction (WF), ethyl acetate fraction (EAF) or *n*-butanol fraction (NBF), 30 min before administration of pentobarbital (30 mg/kg, i.p.). Data are expressed as mean ± SD (*n* = 8). *** *P* < 0.001 vs. Saline and DMSO (for NBF and EAF). Hydro-alcoholic maceration extract (HAME), hydro-alcoholic soxhlet extract (HASE), ethyl acetate fraction (EAF), N-butanol fraction (NBF), and the water fraction (WF).

As illustrated in Figure 4, all three fractions of the HASE (50 mg/kg) of the seeds exhibited sleep prolonging activity compared to NS for WF and compared to DMSO for EAF and NBF (*P* < 0.001 for all cases).

### 3.4. Sleep Latency

**Figure 5:**
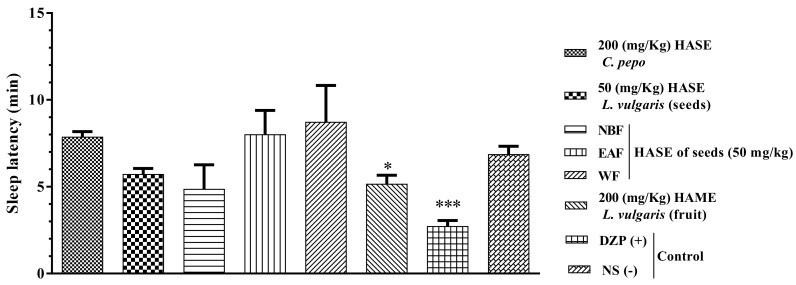
Effects of extracts on sleep latency in mice. The animals were treated with saline, diazepam (3 mg/kg), HASE of *C. pepo* (200 mg/kg) and HASE, water fraction (WF), ethyl acetate fraction (EAF) or N-butanol fraction (NBF) of seeds (50 mg/kg) and HAME of fruit (200 mg/kg) of *L. vulgaris*. Data are expressed as mean ± SD (*n* = 8). * *P* < 0.05 and *** *P* < 0.001 vs. saline. Hydro-alcoholic maceration extract (HAME), hydro-alcoholic soxhlet extract (HASE).

As presented in Figure 5, diazepam notably diminished sleep latency compared to NS (*P* < 0.001). NBF and 200 mg/kg HAME of *L. vulgaris* fruit significantly alleviated the time to before sleep (*P* < 0.05). Furthermore, all fractions and extracts did not lead to decreased sleep latency compared to diazepam.

### 3.5. Sleep Mechanism

**Figure 6:**
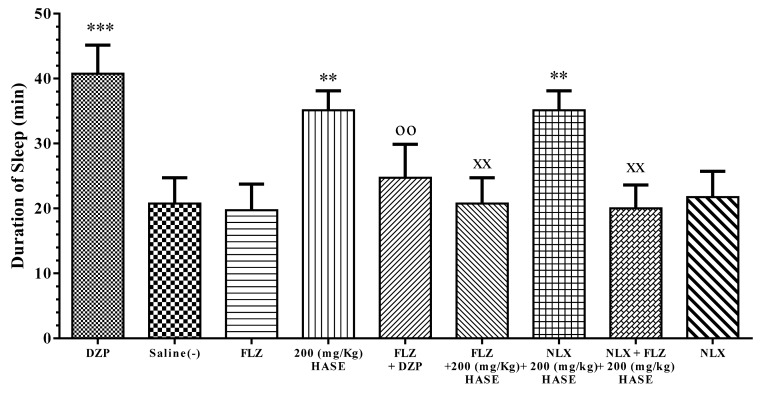
Effect of flumazenil (FLZ) and naloxone (NLX) on the sleep-prolonging effect of *C. pepo*. The animals were treated with saline, 3 mg/kg of diazepam (DZP) or 200 mg/kg of HASE before injection of pentobarbital (30 mg/kg, i.p.). Flumazenil (1 mg/kg i.p.) or/and naloxone (5 mg/kg i.p.) were administrated 30 min before diazepam or HASE. Data are expressed as mean ± SD (*n* = 8). ** *P* < 0.01 and *** *P* < 0.001 vs. saline; ^XX^ *P* < 0.01 vs. HASE; ^oo^ *P* < 0.01 vs. diazepam. Hydro-alcoholic maceration extract (HAME), hydro-alcoholic soxhlet extract (HASE), ethyl acetate fraction (EAF), N-butanol fraction (NBF), and the water fraction (WF).

**Figure 7:**
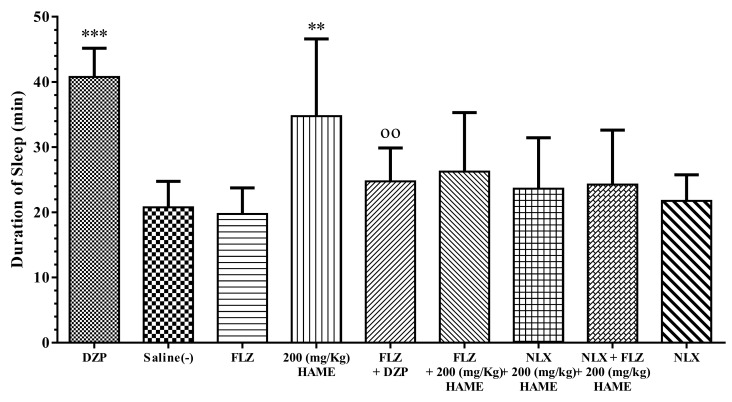
Effect of flumazenil (FLZ) and naloxone (NLX) on the sleep-prolonging effect of *L. vulgaris* fruit. The animals were treated with saline, 3 mg/kg of diazepam (DZP) or 200 mg/kg of HAME before injection of pentobarbital (30 mg/kg, i.p.). Flumazenil (1 mg/kg i.p.) or/and naloxone (5 mg/kg i.p.) were administrated 30 min before diazepam or HAME. Data are expressed as mean ± SD (*n* = 8). ** *P* < 0.01 and *** *P* < 0.001 vs. saline; ^oo^ *P* < 0.01 vs. diazepam. Hydro-alcoholic maceration extract (HAME), hydro-alcoholic soxhlet extract (HASE), ethyl acetate fraction (EAF), N-butanol fraction (NBF), and the water fraction (WF).

**Figure 8:**
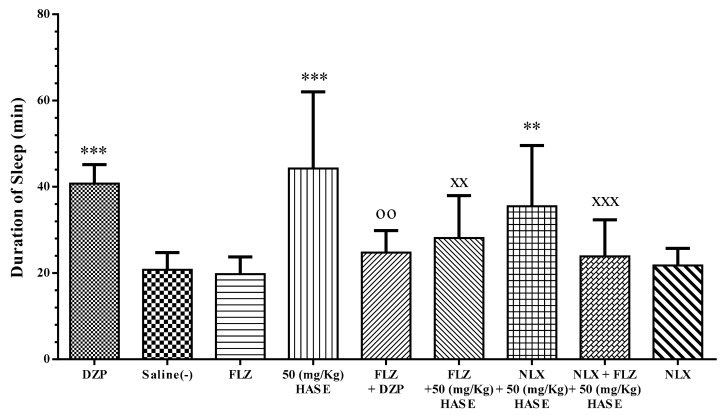
Effect of flumazenil (FLZ) and naloxone (NLX) on the sleep-prolonging effect of *L. vulgaris* seeds. The animals were treated with saline, 3 mg/kg of diazepam (DZP) or 50 mg/kg of HASE before injection of pentobarbital (30 mg/kg, i.p.). Flumazenil (1 mg/kg i.p.) or/and naloxone (5 mg/kg i.p.) were administrated 30 min before diazepam or HASE. Data are expressed as mean ± SD (*n* = 8). ** *P* < 0.01 and *** *P* < 0.001 vs. saline; ^XX^ *P* < 0.01 and ^XXX^ *P* < 0.001 vs. HASE; ^oo^ *P* < 0.01 vs. diazepam. Values are presented as Mean ± SEM (*n* = 8). Hydro-alcoholic maceration extract (HAME), hydro-alcoholic soxhlet extract (HASE), ethyl acetate fraction (EAF), N-butanol fraction (NBF), and the water fraction (WF).

Flumazenil (FLZ), saline and naloxone (NLX) on its own had no effect on the sleep duration induced by pentobarbital. Pretreatment of mice with FLZ decreased the sleep-prolonging effect of diazepam (*P* < 0.01, Figure 6, Figure 7 and Figure 8).

FLZ significantly reversed the sleep-prolonging effect of 200 mg/kg HASE of *C. pepo* in comparison with HASE alone (*P* < 0.01). NLX had no effect on the sleep duration of this extract but pretreatment with NLX + FLZ showed a similar effect to FLZ (Figure 6).

FLZ or/and NLX pretreatment reduced the sleep duration of 200 mg/kg HAME of *L. vulgaris* fruit, but these decreases were not significant (Figure 7).

The dose 50 mg/kg of the HASE of *L. vulgaris* seed, which had the maximum effect on sleep prolongation, was selected to investigate the possible mechanisms (Figure 8). Combination of FLZ and the recent extract significantly reduced sleep duration in comparison with 50 mg/kg HASE alone (*P* < 0.01). Similarly, NLX plus the same extract decreased sleep duration, however this reduction was lower than FLZ plus extract and was not significant. Co-administration of NLX, FLZ and 50 mg/kg of the HASE of *L. vulgaris* seed extremely attenuated the sleep-prolonging effect when compared to the extract alone (*P* < 0.001). The combination of NLX and FLZ possessed a greater reduction than each recent antagonist alone (Figure 8).

### 3.6. Toxicity Assessments

#### 3.6.1. LD_50_ Determination

The highest dose that did not kill any mice and the lowest dose that led to death of one mouse for the HASE of *C. pepo*, *L. vulgaris* seeds and fruit were 3.2 and 6.4 g/kg, 1.6 and 3.2 g/kg and 3.2 and 6.4 g/kg, respectively. The mean of these two doses was considered as the LD_50_ (4.8, 2.4 and 4.8 g/kg, respectively).

#### 3.6.2. Evaluation of the Cytotoxicity Effects of *C. pepo* and *L. vulgaris*

**Figure 9:**
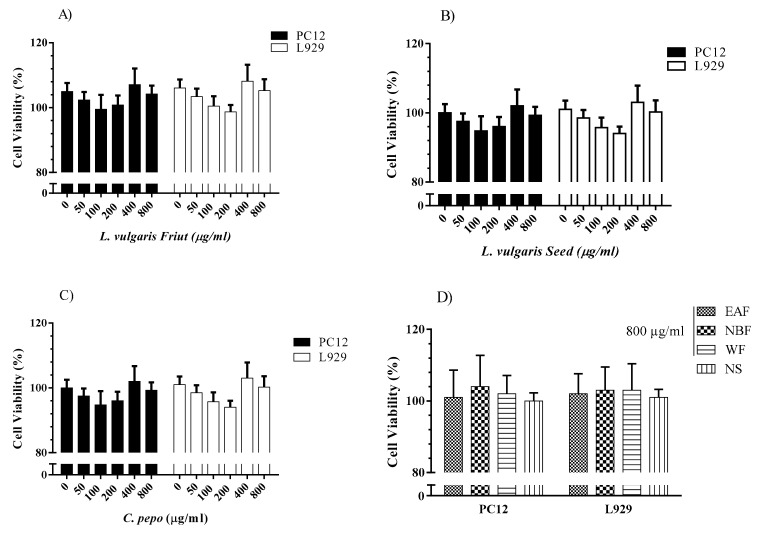
The effect of *L. vulgaris* and *C. pepo* extracts on the viability of PC12 and L929 cells. Data are mean ± SD (*n* = 5). (**A**) hydro-ethanolic macerated extract of *L. vulgaris* fruit; (**B**) hydro-ethanolic soxhlet extract of seeds of *L. vulgaris*; (**C**) hydro-ethanolic soxhlet extract of *C. pepo* and (**D**) 800 µg/mL of water fraction (WF), ethyl acetate fraction (EAF) and *n*-butanol fraction (NBF) of HASE of *L. vulgaris* seeds were examined and compared to saline. Hydro-alcoholic maceration extract (HAME), hydro-alcoholic soxhlet extract (HASE).

The possible cytotoxicity of the extracts was evaluated on PC12 and L929 cells (Figure 9). It was found that up to 800 µg/mL, none of the three extract concentrations decreased the proliferation of PC12 and L929 cells (Figure 9A–C) after 24 h. Furthermore, all three fractions of HASE of L. vulgaris seeds depicted no cytotoxicity on PC12 and L929 cells after 24 h and up to 800 µg/mL (Figure 9D).

## 4. Discussion

Traditional medicine recommends the consumption of *L. vulgaris* and *C. pepo* for different disorders such as hypnotic problems [20,22]. However, in this research we investigated the hypnotic effects of *L. vulgaris* and *C. pepo* for the first time. Our findings showed that the HAME and HASE of *C. pepo* increased sleep duration at a high dose (200 mg/kg). The fruit of *L. vulgaris* also improved sleep duration at a 200 mg/kg dose of HAME. In studying the seeds of *L. vulgaris*, we found that both HAME and HASE also enhanced sleep duration at doses of 50 and 100 mg/kg. Furthermore, all three fractions of the HASE of the seeds potentiated the sleep duration. The neurotoxicity test also revealed that the extracts did not have an effect on cell viability.

Diazepam belongs to the benzodiazepine group, which has a binding site on the GABA receptor type-ionophore complex (GABA_A_) [2,28]. It has been emphasized that diazepam ameliorates the onset of sleep and increases the sleep duration [2]. In our study, we showed that *C. pepo* and *L. vulgaris* potentiate the sedative effect of pentobarbital as well as diazepam. It could be concluded that the hypnotic effects of extracts may be due to their potentiating the GABA system. It has been demonstrated that the inhibitory action of GABA is via chloride channels opening and the hyperpolarization of the membrane, which lead to CNS depression, sedative and hypnotic activity [29]. Therefore, drugs that influence these systems are considered to be important in treating insomnia disorder.

In order to determine the possible role of benzodiazepine or opioid receptors in the hypnotic effects of extracts, flumazenil and naloxone as a selective antagonist of benzodiazepine and opioid receptors were administered, respectively. Our results revealed that FLZ and NLX + FLZ significantly reversed the sleep-prolonging effect of 200 mg/kg HASE of *C. pepo*, while applying NLX had no effect. It could be concluded that BZR plays an important role in hypnotic effects of *C. pepo*. Furthermore, FLZ, NLX and FLZ + NLX diminished the sleep duration of the HAME of the *L. vulgaris* fruit, but not significantly. FLZ and NLX significantly alleviated the sleep duration of 50 mg/kg of the HASE of *L. vulgaris* seed, but the inhibitory effect of FLZ was stronger than NLX. NLX + FLZ showed a greater reduction than each antagonism alone with the seed extract. In fact, our presented findings involve the BZR in the sleep-induction of *L. vulgaris* seeds.

To obtain a better insight into the nature of the compounds that may be responsible for the hypnotic effects of the HASE of *L. vulgaris* seed, the fractionation of extracts according to the polarity of the compounds were performed. For this reason, three fractions were prepared, including: (1) the WF, solubilizing the polar agents and water-soluble plant constituents (e.g., glycosides, quaternary alkaloids, tannins); (2) the EAF, extracting compounds with intermediate polarity; and (3) the NBF, bearing non-polar agents like sterols, alkanes and some terpenoids [2]. Interestingly, we found that all three types of prepared fractions markedly prolonged sleep duration. This means that the HASE of *L. vulgaris* seed has a wide range of non-polar to polar compounds which are responsible for sleep induction. It has been reported that a wide variety of phytochemicals possess sedative–hypnotic effects, including terpenoids (e.g., linalool, eugenol), flavonoids (e.g., vitexin, isovitexin, quercetrin, Luteolin), alkaloids (e.g., rosmarinic acid), steroids (e.g., β-sitosterol) and saponins [30]. Sterols, terpenoids, flavonoids and saponins have been isolated from the *L. vulgaris* extract [20,31]. Previously, phytochemical evaluations of *C. pepo* have also indicated the existence of phenolic and flavonoid compounds in hydro-ethanolic extract [32]. Recent studies have shown that the flavonoids in numerous plant species have anxiolytic and/or anti-depressant activity. This effect has been ascribed to their affinity for the central benzodiazepine receptors [2,28]. Vitexin and isovitexin are two known flavonoids that have been isolated from *L. vulgaris* and that showed protective effects on different models of neurological and psychiatric disorders, including ischemia injury, learning deficit and excitotoxicity [33]. Moreover, it has been shown that these compounds can display anti-convulsant effects on pentylenetetrazole-induced seizures in rats through acting on the benzodiazepine receptor site of GABA_A_ receptor complex [33,34]. In addition, it has been demonstrated that triterpenoids with a steroidal configuration—including ursolic acid [35], oleanolic acid [35,36] and β-sitosterol, as well as β-amyrin [37,38], which is found in *L. vulgaris*, as are other terpenoids—can cross the blood brain barrier due to their lipophilic feature [39,40]. Recently, it has been documented that these mentioned compounds increase sleep duration and decrease sleep onset through acting on the GABA_A_ receptor [35]. Therefore, it could be suggested that flavonoids and triterpenoids of *L. vulgaris* and *C. pepo* contribute to exerting a hypnotic effect of these plants, mostly through benzodiazepine and GABA_A_ receptors, rather than opioid receptors.

To assess the possible toxicity of extracts and fractions, in-vivo and in-vitro tests were performed. Twenty four hour exposure to extracts provided no cytotoxicity against PC12 cells. In fact, this test showed that the extracts’ hypnotic effect and prolongation of sleep duration were not due to their neurotoxicity. Although these extracts showed no in-vivo and in-vitro toxicity in our study, further additional tests using differing methods are needed to establish the safety of the extracts.

## 5. Conclusions

In conclusion, our results revealed that both plants, especially *L. vulgaris* seeds, possess potent sedative–hypnotic properties. Furthermore, the results of the present study showed that extracts exhibit very low toxicity, which is reflected by the high LD50 values for i.p. administration. Therefore, they may be a good candidate for additional pharmacological studies, including anti-convulsant and anti-seizure evaluations. Further chemical and pharmacological analyses of the extracts are needed to isolate and characterize the active components responsible for their sedative effects.
